# A nonparametric approach for quantile regression

**DOI:** 10.1186/s40488-018-0084-9

**Published:** 2018-07-18

**Authors:** Mei Ling Huang, Christine Nguyen

**Affiliations:** 1 Department of Mathematics & Statistics, Brock University, St. Catharines, Ontario, L2S 3A1 Canada; 2 Apotex Inc., Toronto, M9L 1T9 Ontario Canada

**Keywords:** Conditional quantile, Goodness-of-fit, Gumbel’s second kind of bivariate exponential distribution, Nonparametric kernel density estimator, Nonparametric regression, Weighted loss function, primary: 62G32; secondary: 62J05

## Abstract

Quantile regression estimates conditional quantiles and has wide applications in the real world. Estimating high conditional quantiles is an important problem. The regular quantile regression (QR) method often designs a linear or non-linear model, then estimates the coefficients to obtain the estimated conditional quantiles. This approach may be restricted by the linear model setting. To overcome this problem, this paper proposes a direct nonparametric quantile regression method with five-step algorithm. Monte Carlo simulations show good efficiency for the proposed direct QR estimator relative to the regular QR estimator. The paper also investigates two real-world examples of applications by using the proposed method. Studies of the simulations and the examples illustrate that the proposed direct nonparametric quantile regression model fits the data set better than the regular quantile regression method.

## Introduction

It is important to study quantile regression to estimate high conditional quantiles in real-world events Koenker (2005). Some extreme events can cause damages to society: stock market crashes, pipeline failures, large flooding, wildfires, pollution, earth quakes and hurricanes. We wish to estimate high conditional quantiles of a random variable *y* with cumulative distribution function (c.d.f.) *F*(*y*) given a variable vector, **x**=**(***x*_1_**,***x*_2_**,…,***x*_*d*_), and **x**_*p*_=(1,*x*_1_,*x*_2_,…,*x*_*d*_)^*T*^∈*R*^*p*^ where *p*=*d*+1. The *τ*th conditional linear quantile is defined by

1 $$ Q_{y}(\tau |\mathbf{x})=Q_{y}(\tau |x_{1},x_{2},\ldots,x_{d})=F^{-1}(\tau | \mathbf{x}),\text{\ }0<\tau <1.  $$

The traditional quantile regression is concerned with the estimation of the *τ*th conditional quantile regression (QR) of *y* for given **x** which often sets a linear model as

2 $$ Q_{y}(\tau |\mathbf{x})=\mathbf{x}_{p}^{T}\mathbf{\beta }(\tau)=\beta_{0}(\tau)+\beta_{1}(\tau)x_{1}+\cdots +\beta_{d}(\tau)x_{d}, 0<\tau <1,  $$

where **β**(*τ*)=(*β*_0_(*τ*),*β*_1_(*τ*),*β*_2_(*τ*),…,*β*_*d*_(*τ*))^*T*^.

For linear model *(2),* we estimate the coefficient **β**(*τ*)=(*β*_0_(*τ*),*β*_1_(*τ*),*β*_2_(*τ*),…,*β*_*d*_(*τ*))^*T*^∈*R*^*p*^ from a random sample {(*y*_*i*_,**x**_*i*_),*i*=1,…,*n*}, where **x**_*pi*_=(1,*x*_*i*1_,*x*_*i*2_,…,*x*_*id*_)^*T*^ is the *p*-dimensional design vector and *y*_*i*_ is the univariate response variable from a continuous distribution with a c.d.f. *F*(*y*). Koenker and Bassett (1978) proposed an *L*_1_-weighted loss function to obtain estimator $\widehat {\mathbf {\beta }} (\tau)$ by solving

3 $$ \widehat{\mathbf{\beta }}(\tau)=\text{arg}\mathop{\text{min}}\limits_{\mathbf{\beta }(\tau)\in R^{p}}\sum\limits_{i=1}^{n}\rho_{\tau }(y_{i}-\mathbf{x}_{pi}^{T}\mathbf{\beta } (\tau)),\ 0<\tau <1,  $$

where *ρ*_*τ*_ is a loss function, namely

$$\rho_{\tau }(u)=u(\tau -I(u<0))=\left\{ \begin{array}{l} u(\tau -1),u<0; \\ u\tau,\ u\geq 0. \end{array} \right. $$

The linear quantile regression problem can be formulated as a linear program

$$\mathop{\text{min}}\limits_{(\mathbf{\beta }(\tau),\mathbf{u},\mathbf{v})\in R^{p}\times R_{+}^{2n}}\{\tau \mathbf{1}_{n}^{T}\mathbf{u}+(1-\tau)\mathbf{1}_{n}^{T} \mathbf{v}|\mathbf{X\beta }(\tau)+\mathbf{u}-\mathbf{v}=\mathbf{y}\}, $$

where $\mathbf {1}_{n}^{T}$ is an *n*-vector of *1*s, **X** denotes the *n*×*p* design matrix, and **u,v** are *n* × 1 vectors with elements of *u*_*i*_,*v*_*i*_, *i*=1,…,*n*, respectively (Koenker, 2005).

In recent years, studies are looking for efficiency improvements of estimator *(3)* (Yu et al. 2003; Wang and Li 2013; Huang et al. 2015; Huang and Nguyen 2017). The regular linear quantile regression *(2)* needs the estimator $\widehat {\mathbf {\beta }} (\tau)$ in *(3)* for the estimated conditional quantile curves. But this estimated conditional quantile curve may be restricted under the model setting.

Many studies have used nonparametric method of quantile regression in recent years, for example, Chaudhuri (2003), Yu and Jones (1991), Hall et al. (1999) and Yu et al. (2003). Chapter 7 in Keoker (2005) proposed a local polynomial quantile regression (LPQR), and other methods. Also we can see detailed discussions on theory, methodologies and applications in Li and Racine (2007) and Cai (2013).

In order to overcome the limitation of the model setting in *(2)* in this paer we propose a direct nonparametric quantile regression method which uses the ideas of nonparametric kernel density estimation and nonparametric kernel regression. The proposed method is not only different from most other existing nonparametric quantile regression methods, it also overcome thecrossing problem of estimating quantile curves. We like to see if the new method has an improvement relative to the regular linear quantile regression and other nonparametric quantile regression methods, we will do two studies in this paper:

1. Monte Carlo simulations will be performed to confirm the better efficiency of the new direct QR estimator relative to the regular QR estimator and a nonparametric LPQR.

2. The new proposed method will be applied to two real-world examples of extreme events and compared with the linear model in Huang and Nguyen (2017).

In Section 2, we propose a direct nonparametric quantile regression estimator. A relative measure of comparing goodness-of-fit for quantile models is given in Section 3. In Section 4, the results of Monte Carlo simulations generated from Gumbel’s second kind of bivariate exponential distribution Gumbel (1960) show that the proposed direct method produces high efficiencies relative to existing linear QR and LPQR methods. In Section 5, the regular linear quantile regression and the proposed direct quantile regression are applied to two real-life examples: the Buffalo snowfall and CO_2_ emission examples in Huang and Nguyen (2017). The study of these examples illustrate that the proposed direct nonparametric quantile regression model fits the data better than the existing linear quantile regression method.

## Proposed direct nonparametric quantile regression

In this paper, for generality, we ignore the idea of the linear model *(2).* We obtain a direct estimator for true conditional quantile in *(1):*

$$\widehat{Q}_{y}(\tau |\mathbf{x})=\widehat{Q}_{y}(\tau |x_{1},x_{2},\ldots,x_{d})=\widehat{F}^{-1}(\tau |\mathbf{x}), $$

by using local conditional quantile estimator *ξ*_*i*_(*τ*|**x**_*i*_)=*Q*_*y*_(*τ*|**x**_*i*_) based the *i*th point of given random sample, {(*y*_*i*_,**x**_*i*_),*i*= 1,…,*n*}, for **x**_*i*_=(*x*_1*i*_,*x*_2*i*_,…,*x*_*di*_)^*T*^.

We construct the following a five-step algorithm of a direct nonparametric quantile regression:

**Step 1:** Estimate the conditional density of *y* for given **x**=(*x*_1_**,***x*_2_**,…,***x*_*d*_) using a kernel density estimation method (Silverman 1986; Scott 2015):

4 $$ \widehat{f}(y|\mathbf{x})=\frac{\widehat{f}(y,\mathbf{x})}{\widehat{g}(\mathbf{x})},  $$

where $\widehat {f}(y,\mathbf {x})$ is an estimator of the joint density of *y* and **x,** and $\widehat {g}(\mathbf {x)}$ is an estimator of the marginal density of **x**.

A *d*-dimensional kernel density estimator from a random sample **X**_*i*_=(*X*_1*i*_**,***X*_2*i*_,…**,***X*_*di*_), *i*=1,2,…,*n*, from a population **x**=**(***x*_1_**,***x*_2_**,…,***x*_*d*_**)** for joint density *g*(**x**),is given by

$$\widehat{g}(\mathbf{x})=\frac{1}{nh^{d}}\sum\limits_{i=1}^{n}K\left\{ \frac{ \mathbf{x}-\mathbf{X}_{i}}{h}\right\}, $$

where *h*>0 is the bandwidth and the kernel function *K*(**x**) is a function defined for *d*-dimensional **x**=(**x**_1_,**x**_2_,…,**x**_*d*_) which satisfies $\int \limits _{R^{d}}K(\mathbf {x})d \mathbf {x}=1.$

Fukunaga (1972) suggested using

$$\widehat{g}(\mathbf{x})=\frac{(\det \mathbf{S})^{-1/2}}{nh^{d}} \sum\limits_{i=1}^{n}k\left\{ \frac{(\mathbf{x}-\mathbf{X}_{i})^{T}\mathbf{S }^{-1}(\mathbf{x}-\mathbf{X}_{i})}{h^{2}}\right\}, $$

where **S** is the sample covariance matrix of the data, *K* is the normal kernel, the function *k* is

$$k(u)=\left(\frac{1}{2\pi }\right)^{d/2}\exp \left(-\frac{u}{2}\right),\quad k(\mathbf{x}^{T}\mathbf{x)}=K(\mathbf{x})=(2\pi)^{-d/2}\exp \left(- \frac{1}{2}\mathbf{x}^{T}\mathbf{x}\right) \mathbf{.} $$

A plug-in selector of the bandwidth *h*>0 will be given by (Silverman 1986, p. 85) as

5 $$ h_{opt}=\left\{ \int t^{2}K(t)dt\right\}^{-2/(d+2)}\left\{ \int K(t)^{2}dt\right\}^{1/(d+4)}\left\{ \int \left(\nabla^{2}g(\mathbf{x})\right)^{2}d\mathbf{x}\right\}^{-1/(d+4)}n^{-1/(d+4)},  $$

If a multivariate normal kernel is used for smoothing the normal distribution data with unit variance,

$$h_{opt}=\left\{ \frac{4}{d+2}\right\}^{1/(d+4)}n^{-1/(d+4)}. $$

**Step 2:** Estimate the conditional c.d.f. of *y* given **x:**

$$\widehat{F}(y|\mathbf{x})=\int_{-\infty }^{y}\widehat{f}(y|\mathbf{x})dy. $$

**Step 3:** Estimate the local conditional quantile function *ξ*(*τ*|**x**) of *y* given **x** by inverting an estimated conditional c.d.f. $\widehat {F}(y|\mathbf {x})$.

$$\widehat{\xi }(\tau |\mathbf{x})=\widehat{Q_{y}}(\tau |\mathbf{x})=\inf \{y: \widehat{F}(y|\mathbf{x})\geq \tau \}=\widehat{F}^{-1}(\tau |\mathbf{x}). $$

It is difficult to compute a global inverse function $\widehat {\xi }(\tau | \mathbf {x})$ of the kernel estimated conditional c.d.f. $\widehat {F}(y| \mathbf {x})$ which has many terms. To avoid the the computational global difficulties, we estimate the local conditional quantile point *ξ*_*i*_(*τ*|**x**_*i*_) of *y* given **x**_*i*_ by inverting $ \widehat {F}(y|\mathbf {x}_{i})$ at the *i*th data point (*y*_*i*_,**x**_*i*_):

6 $$ \widehat{\xi_{i}}(\tau |\mathbf{x}_{i})=\widehat{Q_{y}}(\tau |\mathbf{x} _{i})=\inf \{y:\widehat{F}(y|\mathbf{x}_{i})\geq \tau \}=\widehat{F} ^{-1}(\tau |\mathbf{x}_{i}),\quad i=1,2,\ldots,n.  $$

Thus, we have *n* points $\left (\mathbf {x}_{i},\widehat {\xi _{i}}(\tau | \mathbf {x}_{i})\right),\;i=1,2,\ldots,n.$

**Step 4:** We propose a direct nonparametric quantile regression estimator for the *τ*th conditional quantile curve of **x** by using Nadaraya-Watson (NW) nonparametric regression estimator (Scott, 2015, p. 242) on $\left (\mathbf {x}_{i},\widehat {\xi _{i}}(\tau | \mathbf {x}_{i})\right),\;i=1,2,\ldots,n:$

7 $$ Q_{D}(\tau |\mathbf{x})=\widehat{\xi }(\tau |\mathbf{x})=\frac{ \sum\limits_{i=1}^{n}K_{\mathbf{h}}\left\{ \mathbf{x}-\mathbf{X} _{i}\right\} \widehat{\xi_{i}}(\tau |\mathbf{x}_{i})}{\sum \limits_{j=1}^{n}K_{\mathbf{h}}\left\{ \mathbf{x}-\mathbf{X}_{j}\right\} } =\sum\limits_{i=1}^{n}W_{h_{\mathbf{x}}}(\mathbf{x},\mathbf{X}_{i}\mathbf{)} \widehat{\xi_{i}}(\tau |\mathbf{x}_{i}),{\quad}0<\tau <1,  $$

where $W_{h_{x}}(\mathbf {x},\mathbf {X}_{i}\mathbf {)}$ is called an equivalent kernel, and **h**=(*h*_1_,…,*h*_*d*_),

$$W_{h_{\mathbf{x}}}(\mathbf{x},\mathbf{X}_{i}\mathbf{)=}\frac{K_{\mathbf{h} }\left\{ \mathbf{x}-\mathbf{X}_{i}\right\} }{\sum\limits_{j=1}^{n}K_{ \mathbf{h}}\left\{ \mathbf{x}-\mathbf{X}_{j}\right\} },\quad i=1,2,\ldots,n, $$

where

$$K_{\mathbf{h}}\left\{ \mathbf{x}-\mathbf{X}_{i}\right\} =\frac{1}{ nh_{1}\ldots{h}_{d}}\prod\limits_{j=1}^{d}K\left(\frac{x-x_{ij}}{h_{j}}\right),\quad i=1,\ldots,n, $$

where *K* is the kernel function, and *h*_*j*_>0 is the bandwidth for the *j* th dimension.

The new point of *(7)* is that it uses Step 3’s *(6)*numerical results: *n* points $\left (\mathbf {x}_{i},\widehat {\xi _{i}}(\tau |\mathbf {x}_{i})\right),\;i=1,2,\ldots,n,$ to estimate a conditional mean curve of the *τ*th quantile function based on these *n* points, then smoothes these *n* points out.

In this paper, for the kernel regression, we use *K* which is the standard normal kernel. Similar as formula*(5)*, we use the optimal bandwidth for the *j*th dimension (Silverman 1986, p.40),

8 $$ {} h_{j,opt}\,=\,\left\{ \int t^{2}K(t)dt\right\}^{-2/5}\left\{ \int K(t)^{2}dt\right\}^{1/5}\left\{ \int \left(\nabla^{2}\widehat{g_{j}} (x_{j})\right)^{2}d\mathbf{x}_{j}\right\}^{-1/5}n^{-1/5},\quad j\,=\,1,\ldots,d,  $$

where $\widehat {g}_{j}(x_{j})$ is the estimated the *j*th dimensional marginal density of *x*_*j*_ in **x**=(*x*_1_,*x*_2_,…**,***x*_*d*_), *n* is the sample size of the random sample in *(4)*.

**Step 5:** Check all procedures, and make any necessary adjustments.

## Comparison of goodness-of-fit on quantile regression models

In order to compare the regular QR estimator in *(3)*and the direct nonparametric QR estimator in *(7),* we extend the idea of measuring goodness-of-fit by Koenker and Machado (1999). We suggest using a Relative *R*(*τ*), 0<*τ*<1, which is defined as

9 $$ Relative\text{ }R(\tau)=1-\frac{V_{D}(\tau)}{V_{R}(\tau)},\quad -1\leq R(\tau)\leq 1,\quad \text{where}  $$

$$V_{D}(\tau)=\sum_{y_{i}\geq Q_{D}(\tau |\mathbf{x}_{i})}\frac{\tau }{n} \left\vert y_{i}-Q_{D}(\tau |\mathbf{x}_{i})\right\vert +\sum_{y_{i}<Q_{D}(\tau |\mathbf{x}_{i})}\frac{(1-\tau)}{n}\left\vert y_{i}-Q_{D}(\tau |\mathbf{x}_{i})\right\vert, $$

where *Q*_*D*_(*τ*|**x**_*i*_) is obtained by *(7),* and

$$V_{R}(\tau)=\sum_{y_{i}\geq \mathbf{x}_{i}^{T}\widehat{\mathbf{\beta }} (\tau)}\frac{\tau }{n}\left\vert y_{i}-\mathbf{x}_{i}^{T}\widehat{\mathbf{ \beta }}(\tau)\right\vert +\sum_{y_{i}<\mathbf{x}_{i}^{T}\widehat{\mathbf{ \beta }}(\tau)}\frac{(1-\tau)}{n}\left\vert y_{i}-\mathbf{x}_{i}^{T} \widehat{\mathbf{\beta }}(\tau)\right\vert, $$

where $\widehat {\mathbf {\beta }}(\tau)$ is given by *(3).*

## Simulations

For investigating the proposed direct nonparametric quantile regression estimator in *(7),* in this Section, Monte Carlo simulations are performed. We generate *m* random samples with size *n* each from the second kind of Gumbel’s bivariate exponential distribution Gumbel (1960) which has a non-linear conditional quantile function of *y* given *x* in *(11).* It has c.d.f. *F*(*x*,*y*) and density function *f*(*x*,*y*) in *(10)* :

10 $$ F(x,y)=(1-e^{-x})(1-e^{-y})(1+\alpha e^{-(x+y)}),\;x\geq 0,\;y\geq 0,\;\alpha >0,  $$

$$f(x,y)=e^{-(x+y)}(1+\alpha (2e^{-x}-1)(2e^{-y}-1)),\;x\geq 0,\;y\geq 0,\;\alpha >0. $$

The conditional density of *y* for given *x* is

$$f(y|x)=e^{-y}(1+\alpha (2e^{-x}-1)(2e^{-y}-1)),\;x\geq 0,\;y\geq 0,\;\alpha >0. $$

The conditional c.d.f. of *y* for given *x* is

$$F(y|x)=e^{-y}(\alpha (2e^{-x}-1)(1-e^{-y})-1)+1,\;x\geq 0,\;y\geq 0,\;\alpha >0. $$

The true *τ*th conditional quantile function of *y* given *x* of *(10)* is

11 $$\begin{array}{@{}rcl@{}} \xi (\tau |x)\,=\,Q_{y}(\tau |x)\,=\,\ln \left(\frac{2\alpha (2e^{-x}-1)}{\alpha (2e^{-x}\,-\,1)\,-\,1\,+\,\sqrt{(\alpha (2e^{-x}\,-\,1)\,+\,1)^{2}-4\alpha \tau (2e^{-x}-1)}} \right), \\ x\geq 0,\;\alpha >0,\;0<\tau <1. && \notag \end{array} $$

**Fig. 1:**
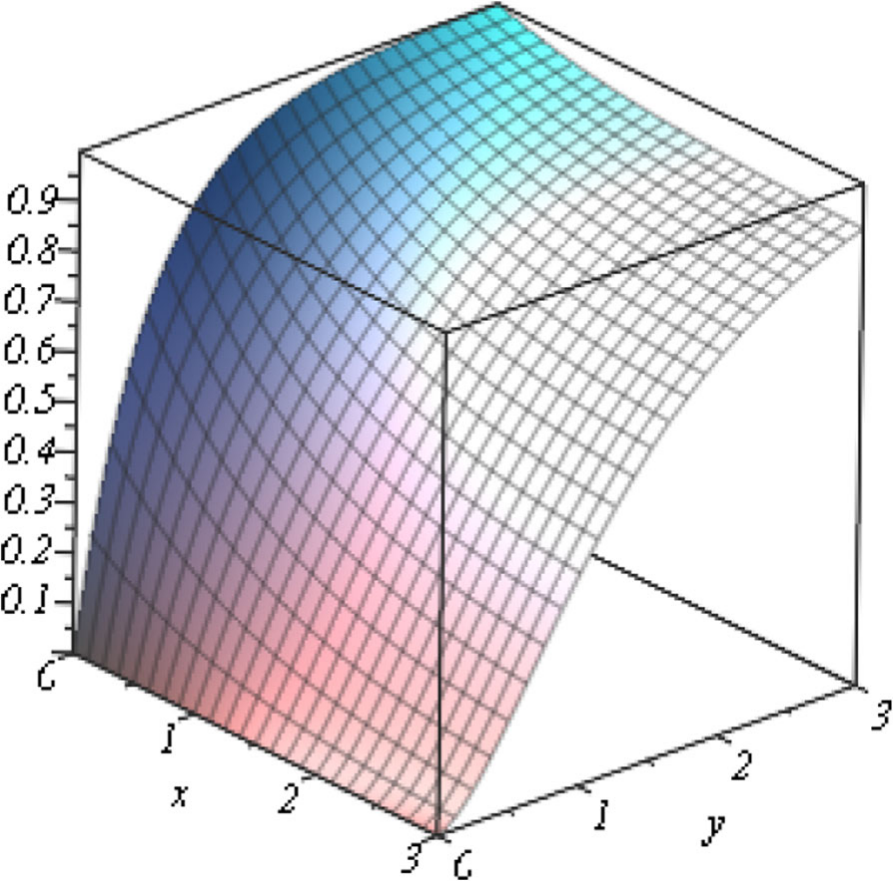
The c.d.f. of Gumbel’s Second kind of bivariate exponential distribution with *α*=1

Letting *α*=1, the c.d.f. in *(10)* is in Fig. 1.

We use three quantile regression methods:

1. The regular quantile regression *Q*_*R*_(*τ*|*x*) estimation based on *(3):*

12 $$ Q_{R}(\tau |x)=\widehat{\beta }_{0}(\tau)+\widehat{\beta }_{1}(\tau)x.\quad 0<\tau <1  $$

2. The first-order linear polynomials Quantile Regression (LPQR) *Q*_*LP*_(*τ*|*x*) (Chaudhuri 1991, Keoker 2005, Yu and Jones 1998), for *z* in a neighborhood of *x*,

13 $$ Q_{LP}(\tau |x)=\widehat{a}_{0}(\tau,x)+\widehat{a}_{1}(\tau,x)(z-x).\quad 0<\tau <1,  $$

where

$$\widehat{\mathbf{a}}(\tau,x)=\arg \min_{\mathbf{\beta }(\tau)\in R^{p}}\sum\limits_{i=1}^{n}\rho_{\tau }(y_{i}-a_{0}(\tau,x)-a_{1}(\tau,x)(x_{i}-x))K\left(\frac{x-x_{i}}{h}\right),\quad 0<\tau <1, $$

here **a**(*τ*,*x*)=(*a*_0_(*τ*,*x*),*a*_1_(*τ*,*x*))^*T*^,*h* and *K* are the bandwidth and kernel function. the LPQR can be computed by the *R* package ‘quantreg’ Koenker (2018).

3. The direct nonparametric quantile regression *Q*_*D*_(*τ*|*x*) estimation based on *(7)*

14 $$ Q_{D}(\tau |x)=\sum\limits_{i=1}^{n}W_{h_{\mathbf{x}}}(\mathbf{x},\mathbf{X} _{i}\mathbf{)}\widehat{\xi_{i}}(\tau |x_{i}),\quad 0<\tau <1,  $$

where $\widehat {\xi _{i}}(\tau |x_{i})$ is obtained by *(6),*$W_{h_{ \mathbf {x}}}(\mathbf {x},\mathbf {X}_{i}\mathbf {)}$ is given by *(7).*

For each method, we generate size *n*=100,*m*=100 samples. *Q*_*R*,*i*_(*τ*|*x*),*Q*_*LP*,*i*_(*τ*|*x*) and *Q*_*D*,*i*_(*τ*|*x*), *i*=1,2,…,*m*, are estimated in the *i*th sample. Let *α*=1 in *(11).* Then the true *τ*th conditional quantile is

15 $$ {} \xi (\tau |x)=Q_{y}(\tau |x)=\ln \left(\frac{2e^{-x}-1}{e^{-x}-1+\sqrt{ e^{-2x}-\tau (2e^{-x}-1)}}\right),\;x\geq 0,\;\alpha >0,\;0<\tau <1.  $$

The simulation mean squared errors (SMSEs) of the estimators *(12)*, *(13)* and *(14)* are:

16 $$\begin{array}{@{}rcl@{}} SMSE(Q_{R}(\tau |x)) &=&\frac{1}{m}\sum\limits_{i=1}^{m}\int_{0}^{N}(Q_{R,i}(\tau |x)-Q_{y}(\tau |x))^{2}dx; \end{array} $$

17 $$\begin{array}{@{}rcl@{}} SMSE(Q_{LP}(\tau |x)) &=&\frac{1}{m}\sum\limits_{i=1}^{m}\int_{0}^{N}(Q_{LP,i}(\tau |x)-Q_{y}(\tau |x))^{2}dx, \end{array} $$

18 $$\begin{array}{@{}rcl@{}} SMSE(Q_{D}(\tau |x)) &=&\frac{1}{m}\sum\limits_{i=1}^{m}\int_{0}^{N}(Q_{D,i}(\tau |x)-Q_{y}(\tau |x))^{2}dx, \end{array} $$

where the true *τ*th conditional quantile *Q*_*y*_(*τ*|*x*) is defined in *(15)*. *N* is a finite *x* value such that the c.d.f. in *(10)* *F*(*N*,*N*)≈1. We take *N*=6 and the simulation efficiencies (SEFFs) are given by

$$SEFF(Q_{LP}(\tau |x))=\frac{SMSE(Q_{R}(\tau |x))}{SMSE(Q_{LP}(\tau |x))},\quad SEFF(Q_{D}(\tau |x))=\frac{SMSE(Q_{R}(\tau |x))}{SMSE(Q_{D}(\tau |x))}, $$

where *SMSE*(*Q*_*R*_(*τ*|*x*)),*SMSE*(*Q*_*LP*_(*τ*|*x*)) and *SMSE*(*Q*_*D*_(*τ*|*x*)) are defined in *(16), (17)* and *(18),* respectively.

Table 1 shows that all of the *SEFF*(*Q*_*D*_(*τ*|*x*)) are larger than 1 when *τ*=0.95,…, 0.99.

**Table 1:** Simulation Mean Square Errors (SMSEs) and Efficiencies (SEFFs) of Estimating *Q*_*y*_(*τ*|*x*),*m*=100,*n*=100,*N*=6.

*τ*	0.95	0.96	0.97	0.98	0.99
*SMSE*(*Q*_*R*_(*τ*|*x*))	22.091	26.632	28.982	42.725	73.340
*SMSE*(*Q*_*LP*_(*τ*|*x*))	8.160	9.667	11.074	15.080	23.734
*SMSE*(*Q*_*D*_(*τ*|*x*))	5.161	6.630	6.552	8.850	11.596
Efficiency					
*SEFF*(*Q*_*LP*_(*τ*|*x*))	**2.7072**	**2.7449**	**2.6171**	**2.8332**	**3.0901**
*SEFF*(*Q*_*D*_(*τ*|*x*))	**4.2804**	**4.0169**	**4.4234**	**4.8278**	**6.3246**

**Fig. 2:**
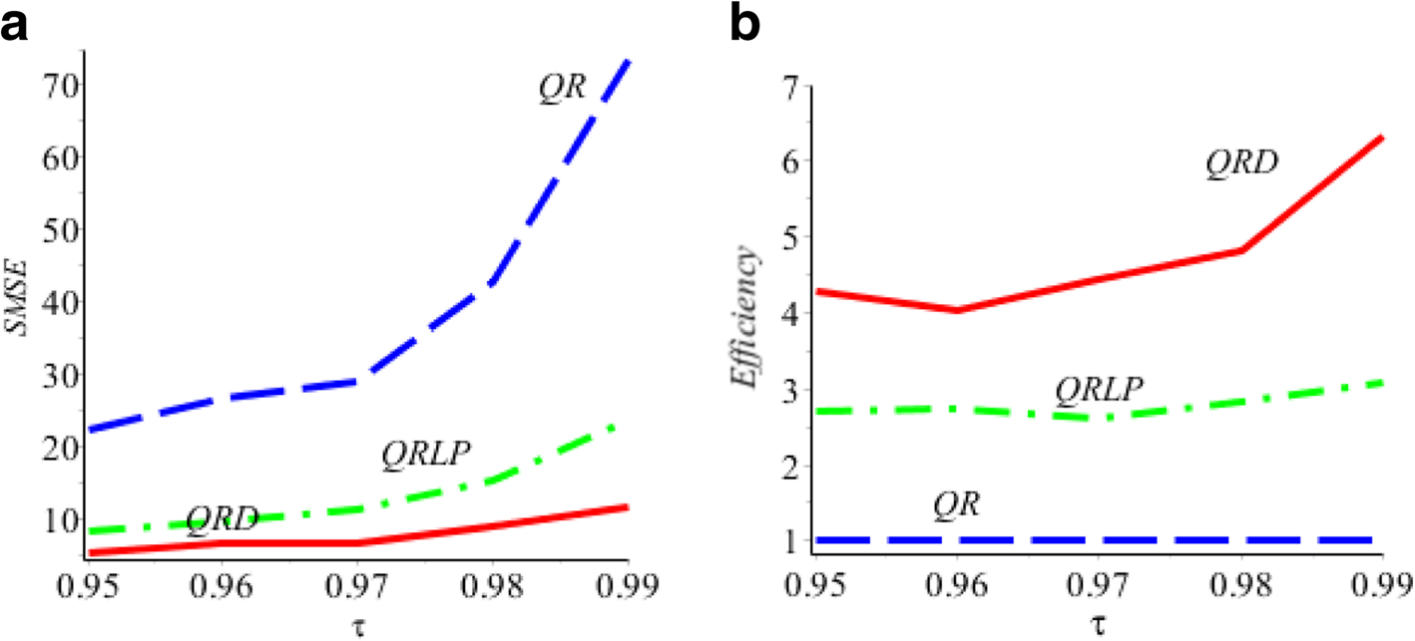
**a** *SMSE*(*Q*_*D*_(*τ*)) is the red solid line, *SMSE*(*Q*_*LP*_(*τ*)) is the green dash-dot line, *SMSE*(*Q*_*R*_(*τ*|*x*)) is the blue dash line. **b** *SEFF*(*Q*_*D*_(*τ*|*x*)) is the red solid line, *SEFF*(*Q*_*LP*_(*τ*|*x*)) is the green dash-dot line, *SEFF*(*Q*_*R*_(*τ*|*x*))≡1 is blue dash line

Figure 2 compares the *SMSE*(*Q*_*R*_(*τ*|*x*)),*SMSE*(*Q*_*LP*_(*τ*|*x*)) with the *SMSE*(*Q*_*D*_(*τ*|*x*)) for *τ*=0.95,…,0.99. It demonstrates that all *SMSE*(*Q*_*D*_(*τ*|*x*)) have smaller values than both *SMSE*(*Q*_*LP*_(*τ*|*x*)) and *SMSE*(*Q*_*R*_(*τ*|*x*)), thus, the simulation results show that the proposed estimator *Q*_*D*_(*τ*|*x*) is more efficient relative to the regular linear estimator *Q*_*R*_(*τ*|*x*) and nonparametric local polynomial estimator *Q*_*D*_(*τ*|*x*).

**Fig. 3:**
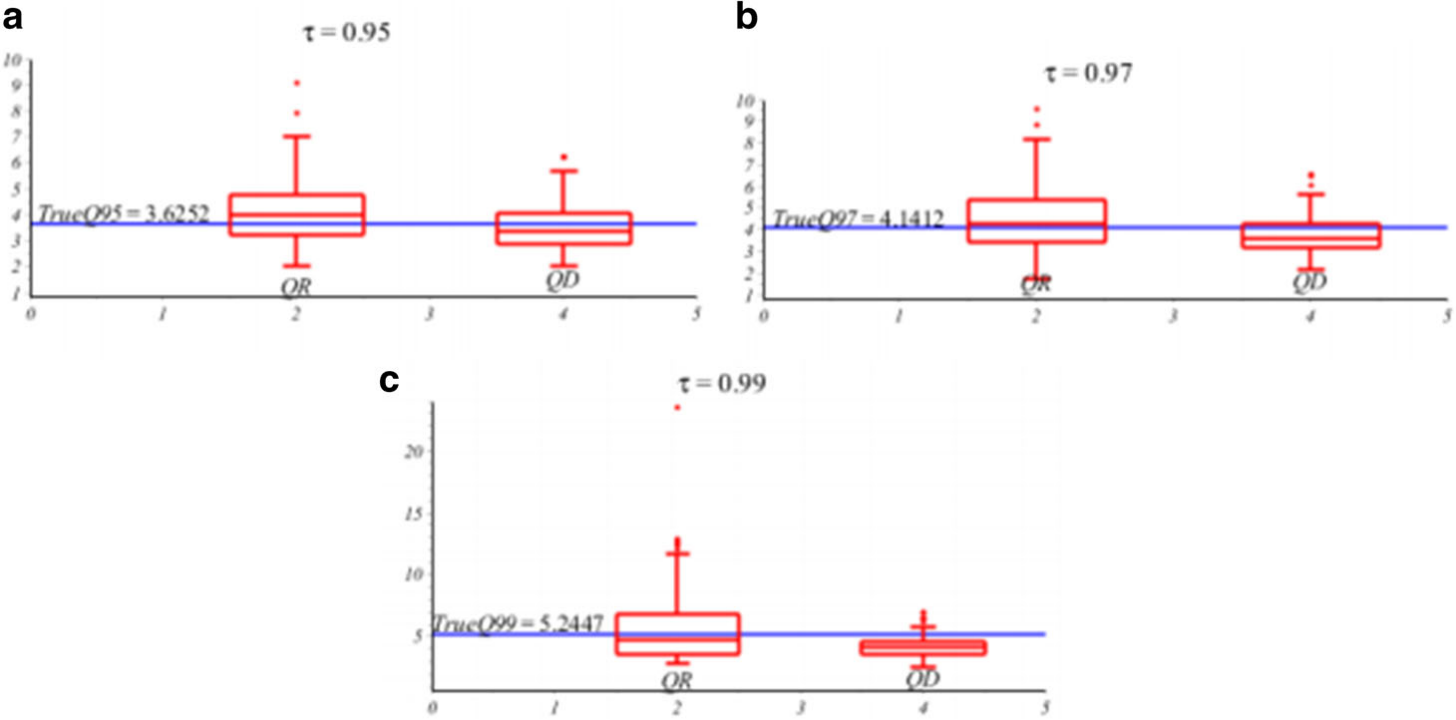
Box plots for (**a**) *τ*=0.95th quantile curves; (**b**) *τ*=0.97th quantile curves; (**c**) *τ*=0.99th quantile curves. The true conditional quantile lines are in blue

**Fig. 4:**
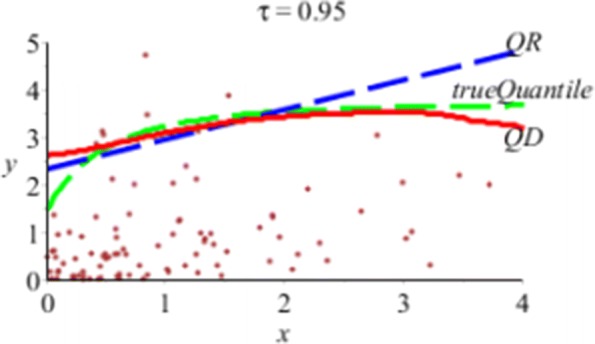
In *n*=100,*m*=100, *τ*=0.95 simulations, the true Quantile-green dash; average regular QR-blue dash; average direct QD-red solid

Next, we compare *Q*_*D*_(*τ*|*x*) and *Q*_*R*_(*τ*|*x*) in Figs. 3 and 4.

Figure 3 shows the boxplots of *Q*_*R*_(*τ*|*x*) and *Q*_*D*_(*τ*|*x*) for *τ*=0.95,0.97, and 0.99.(The true conditional quantiles are in blue line). The *Q*_*D*_(*τ*|*x*) has much smaller variance than *Q*_*R*_(*τ*|*x*)*s*.

Figure 4 shows the average curves of the 100 estimated *τ*=0.95th quantile curves of *Q*_*R*_(*τ*|*x*) (in blue dash line) and that of *Q*_*D*_(*τ*|*x*) (in red solid). The average *Q*_*D*_(*τ*|*x*) curve is much closer than *Q*_*R*_(*τ*|*x*) to the true quantile curve (in green dash).

From the overall results of the simulation, we can conclude that Table 1 and Figs. 2, 3, and 4 show that for *τ*=0.95,…,0.99, the proposed direct estimator *Q*_*D*_(*τ*|*x*) in *(7)* is more efficient relative to the regular regression *Q*_*R*_(*τ*|*x*) in *(2)* and a nonparametric LPQR in *(13).*

## Real examples of applications

In this section, we apply the following two regression models to the Buffalo snowfall and CO_2_ emission examples in Huang and Nguyen (2017):

1. The regular quantile regression *Q*_*R*_(*τ*|**x**) in model *(2)*usingestimator $\widehat {\beta }(\tau)$ in *(3)*;

2. The direct nonparametric quantile regression *Q*_*D*_(*τ*|**x**) in *(7).*

### 5.1 Buffalo snowfall example

Huang and Nguyen (2017) used the following linear second order polynomial quantile regression model for this example (National Weather Service Forecast Office 2017):

$$Q_{y}(\tau |x)=\beta_{0}(\tau)+\beta_{1}(\tau)x+\beta_{2}(\tau)x^{2}, $$

where *y* represents the total snowfall (*cm*) and *x* represents the maximum temperature (°*C*).

**Fig. 5:**
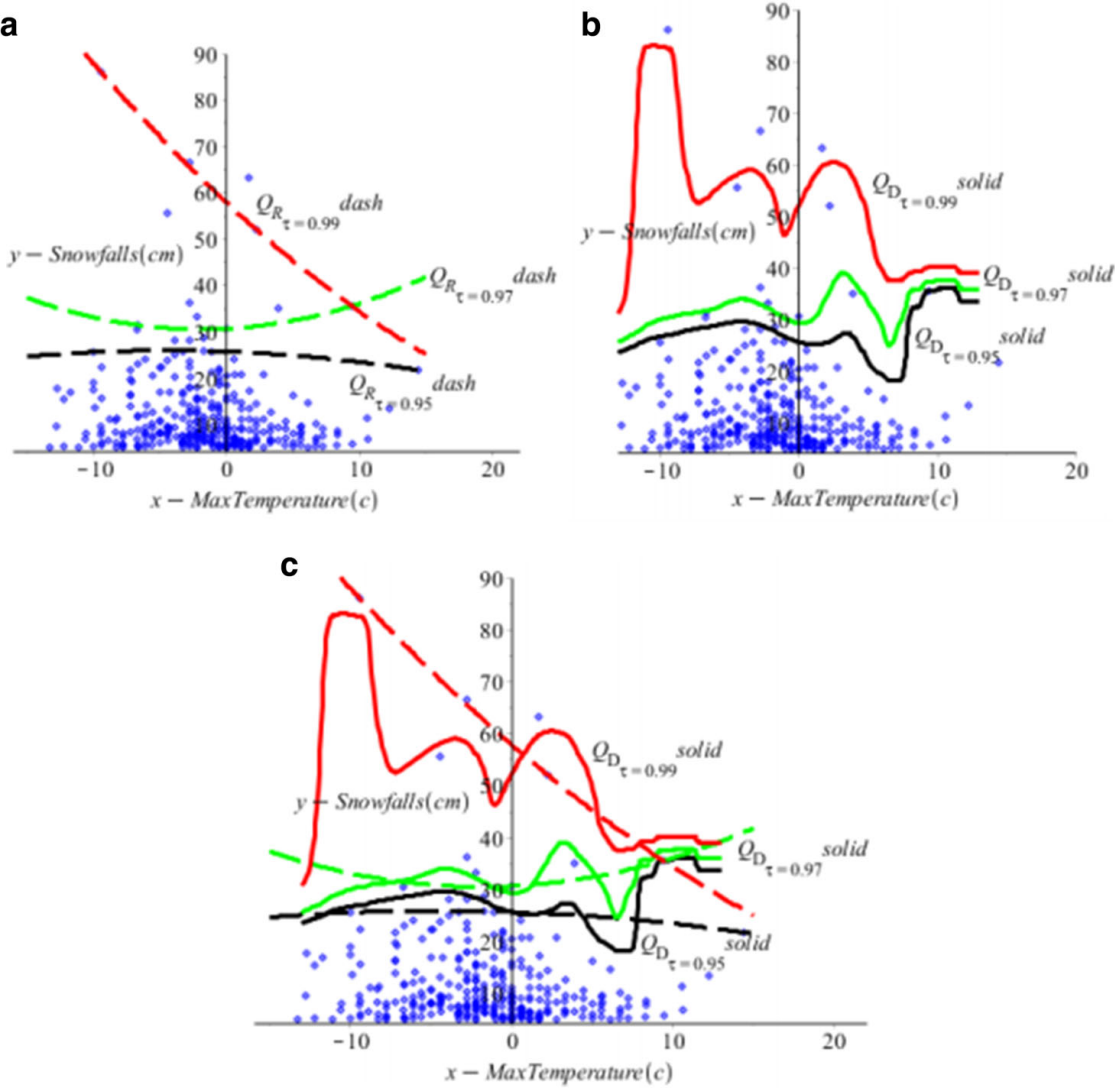
For Buffalo snowfall example, data − blue, *n*=316, (**a**) Regular *Q*_*R*_− dash; (**b**) Direct *Q*_*D*_− solid; (**c**) Both of the Regular *Q*_*R*_ and Direct *Q*_*D*_ in a plot at *τ*=0.95 in black, *τ*=0.97 in green and *τ*=0.99 in red

In this paper we use the proposed five-step algorithm in Section 2 to obtain the new direct nonparametric quantile estimator *Q*_*D*_(*τ*|**x**) in *(7).* We compare the new estimator *Q*_*D*_(*τ*|**x**) with the regular quantile estimator *Q*_*R*_(*τ*|**x**) in Huang and Nguyen (2017). Table 2 and Fig. 5 show the difference of values of two estimators. Figure 5a, b and c show the scatter plot of the daily snowfall vs. maximum temperature with the fitted *Q*_*R*_, and *Q*_*D*_ quantile curves at *τ*= 0,95, 0.97 and 0.99. It is interesting to see that the *Q*_*D*_ curves appear to follow the data patterns closer than the *Q*_*R*_ curves.

**Table 2:** Buffalo Daily Snowfalls (cm) at High Quantiles Using *Q*_*R*_ and *Q*_*D*_

	*τ*=0.97		*τ*=0.99	
Temperature (°*C*)	*Q*_*R*_	*Q*_*D*_	*Q*_*R*_	*Q*_*D*_
-15	37.38	25.49	105.46	60.64
-10	33.19	30.23	87.95	62.98
-5	30.98	33.33	72.08	56.54
0	30.73	29.89	57.86	54.56
5	32.47	33.27	45.29	52.39
10	36.17	37.34	34.36	43.04

Table 2 lists the estimated Buffalo snowfall quantile values at a given maximum temperature for *τ*= 0.97 and 0.99. It demonstrates that when quantiles are at high *τ*, the *Q*_*D*_ gives greater variety of snowfall predictions than the *Q*_*R*_. The relationship of snowfall and max-temperature is not necessarily linear.

**Fig. 6:**
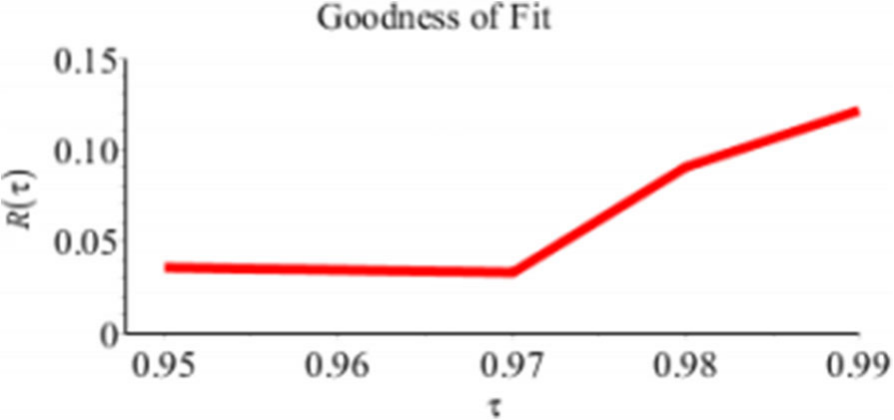
Relative *R*_*τ*_ of *Q*_*D*_ relative to *Q*_*R*_ for the Buffalo snowfall example

Figure 6 and Table 3 show the values of the Relative *R*(*τ*) in *(9)* for given *τ*=0.95,…,0.99. We note that *R*(*τ*)>0 which means that *V*_*D*_(*τ*)<*V*_*R*_(*τ*) and *Q*_*D*_ is a better fit to the data than *Q*_*R*_.

**Table 3:** Relative *R*(*τ*) Values for the Buffalo Snowfall Example

	*τ*=0.95	*τ*=0.96	*τ*=0.97	*τ*=0.98	*τ*=0.99
Relative *R*(*τ*)	0.0359	0.0346	0.0324	0.0903	0.1206

Figure 5c shows that the proposed direct nonparametric quantile regression *Q*_*D*_ predicts that for moderate temperatures, such as 5°*C* to 10°*C*, it is likely to have smaller but varied snowfalls in Buffalo than the regular *Q*_*D*_ predicts. For temperature over 10°*C*, the *Q*_*D*_ predicts a much higher value snow amount than the regular *Q*_*R*_ predicts. On another side, for very low temperatures, such as − 15°*C* to 0°*C*, the *Q*_*D*_ and *Q*_*R*_ both predict more likely to have extreme heavy snowfalls that may cause damage. Thus prediction of heavy snowfalls is related to cold weather forecasts. But the prediction snowfalls related to temperature from the *Q*_*D*_ is not as a simple linear relationship as *Q*_*R*_ predicts. We also note that lots of snow occurred between - 5°*C* to 0°*C*; the predictions form the *Q*_*D*_ are reflecting this fact and give varied predictions.

### 5.2 CO_2_ emission example

Huang and Nguyen (2017) used the linear quantile regression model for this example:

$$Q_{y}(\tau |x_{1},x_{2})=\beta_{0}(\tau)+\beta_{1}(\tau)x_{1}+\beta_{2}(\tau)x_{2}, $$

where y represents CO_2_ emission (tonnes) per capita, *x*_1_ represents ln of gross domestic product (GPD) (US $), per capita and *x*_2_ represents ln of electricity consumption (E.C.) (kilowatts) per capita (Carbon Dioxide Information Analysis Centre (2017)).

**Fig. 7:**
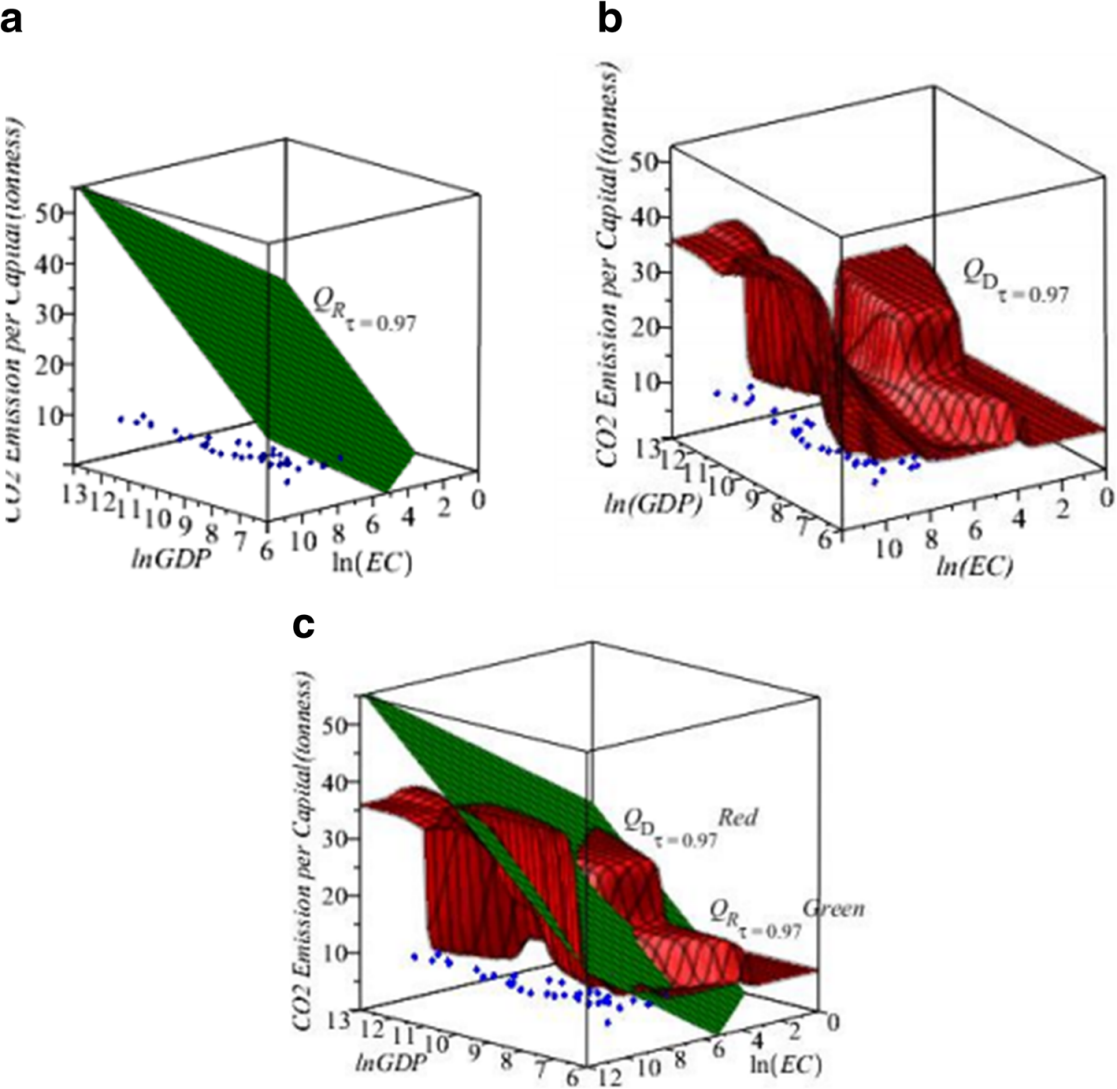
3D Plots for CO_2_ Emission, data − blue, *n*=123, (**a**) Regular *Q*_*R*_− green at *τ*=0.97; (**b**) Direct *Q*_*D*_− red at *τ*=0.97; (**c**) Regular *Q*_*R*_−green and Direct *Q*_*D*_−red in a plot at *τ*=0.97

**Fig. 8:**
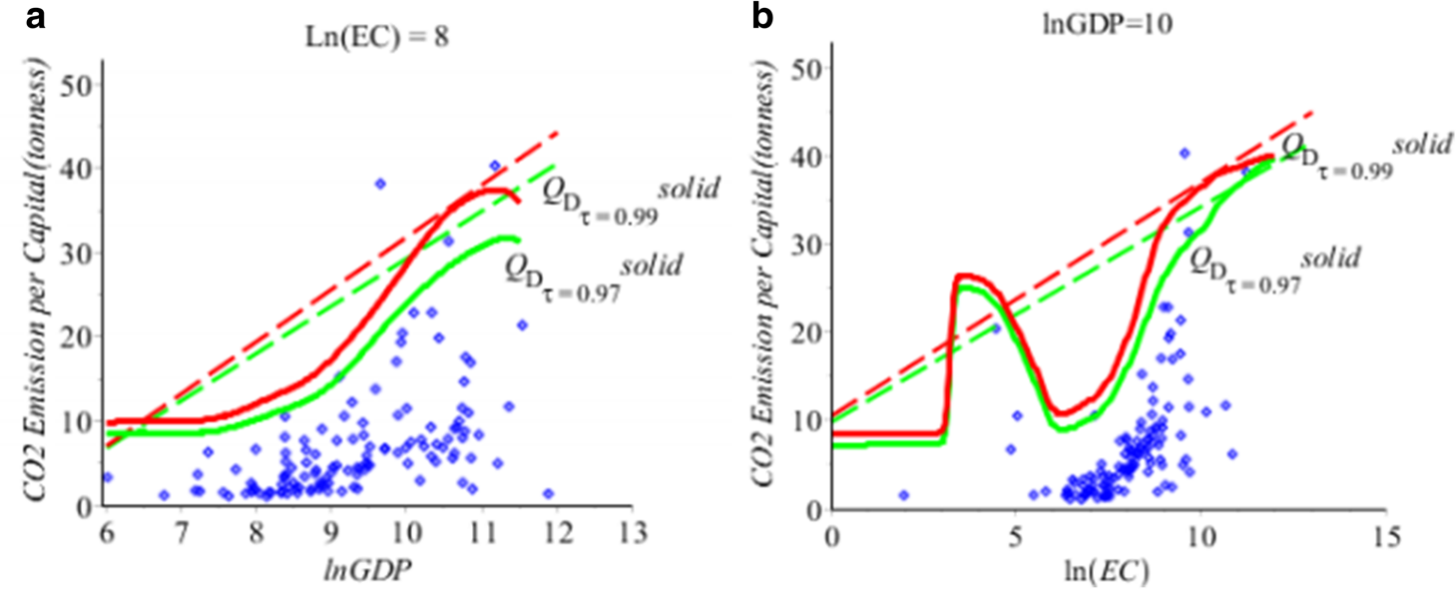
2D plots for CO_2_ Emission, data − blue, *n*=123, (**a**) Regular *Q*_*R*_ (in dash) and direct *Q*_*D*_ (in solid) of the CO_2_ emission vs ln(GDP) when the country’s E.C. is 2980.96 kilowatts at *τ*=0.97 (green) and 0.99 (red). (**b**) Regular *Q*_*R*_ (in dash) and direct *Q*_*D*_ (in solid) of the CO_2_ emission vs ln(E.C.) when the country’s GDP is $13,359.73 at *τ*=0.97 (green) and 0.99 (red)

Similar as in the Buffalo Snowfall example in Subsection 5.1, we use the proposed five-step algorithm in Section 2 to obtain the new direct nonparametric quantile estimator *Q*_*D*_(*τ*|**x**) in *(7).* We compare the new estimator *Q*_*D*_(*τ*|**x**) with the regular quantile estimator *Q*_*R*_(*τ*|**x**) in Huang and Nguyen (2017). Figures 7, 8 and Tables 4, 5 show the differences of the values of two estimators. Figure 7a shows the 3D scatter plot of CO_2_ emission vs ln(GDP) and ln(EC) with the fitted regular *Q*_*R*_ surface at *τ*=0.97. Figure 7b shows the 3D scatter plot of CO_2_ emission vs ln(GDP) and ln(EC) with the fitted direct *Q*_*D*_ surface at *τ*=0.97. Figure 7c shows the 3D scatter plot with both the regular *Q*_*R*_ (green) and direct *Q*_*D*_ (red) quantile surfaces of CO_2_ emission vs the ln(GDP) and ln(E.C.) at *τ*=0.97. It is interesting to see the difference between the *Q*_*R*_ and *Q*_*D*_ quantile surfaces.

**Table 4:** CO_2_ Emission per capita at high quantiles given ln(GDP) estimators *Q*_*R*_ and *Q*_*D*_

	*τ*=0.97	
ln of GDP per capita ($)	*Q*_*R*_	*Q*_*D*_
7.5	15.2181	8.8737
8	18.0437	10.1949
8.5	20.8693	11.7828
9	23.6950	14.4143
9.5	26.5206	19.0458
10	29.3462	24.0338
10.5	32.1718	27.9596
11	34.9975	31.1097
11.5	37.8231	30.7696
12	40.6487	31.2366

2980.96 Kilowatts of Electricity Consumed per capita

**Table 5:** CO_2_ emission per capita at high quantiles given ln(E.C.) estimators *Q*_*R*_ and *Q*_*D*_

ln of Electricity Consumption	*τ*=0.97	
per capita (kilowatts)	*Q*_*R*_	*Q*_*D*_
0	6.9775	7.1919
2	11.8632	7.2759
4	16.7490	24.6924
6	21.6348	9.5560
8	26.5206	15.9569
10	31.4064	31.5634
12	36.2921	39.6481

GDP per capita of 13,359.73

We may see the *Q*_*R*_ and *Q*_*D*_ quantile curves more cleanly in 2D plots. Figure 8a shows the 2D scatter plot of CO_2_ emission vs ln(GDP) when the country’s E.C. is 2980.96 kilowatts with the fitted regular *Q*_*R*_ and direct *Q*_*D*_ curves at at *τ*=0.97. Figure 8b shows the 2D scatter plot of CO_2_ emission vs ln(E.C.) when the country’s GDP is $13,359.73 with the fitted regular *Q*_*R*_ and direct *Q*_*D*_ curves at at *τ*=0.97. We note that the *Q*_*R*_ and *Q*_*D*_ quantile regression curves appear to fit the data. In general, the *Q*_*D*_ curves follow the data patterns closer than *Q*_*R*_ quantile lines, and the *Q*_*D*_ produces different estimated CO _2_ emissions than the *Q*_*R*_ estimated at high quantiles. In Fig. 7, it is interesting to see that the *Q*_*D*_ conditional quantile surfaces are not linear as the linear planes of the *Q*_*R*_.

Tables 4 and 5 provide details of the estimated high quantiles about countries’ CO_2_ emission at *τ*=0.97 when the countries consume 2980.96 kilowatts of electricity and have a GDP of $13,359.73, respectively.

**Fig. 9:**
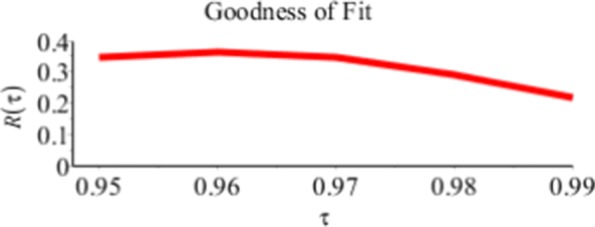
Relative *R*(*τ*) of *Q*_*D*_ relative to *Q*_*R*_ for the CO_2_ emission example

Figure 9 and Table 6 show the Relative *R*(*τ*) in *(9),* for *τ*=0.95,…,0.99. All values of Relative *R*(*τ*) are larger than 0, which signifies that *V*_*D*_(*τ*)<*V*_*R*_(*τ*) and it also suggests that the direct quantile regression estimator *Q*_*D*_ is a better fit to the CO _2_ emission data than the regular quantile regression estimator *Q*_*R*_.

**Table 6:** Relative *R*(*τ*) values for CO_2_ emission example

	*τ*=0.95	*τ*=0.96	*τ*=0.97	*τ*=0.98	*τ*=0.99
Relative *R*(*τ*)	0.3480	0.3612	0.3494	0.2895	0.2151

Over all, it is interesting to see that the proposed direct estimator *Q*_*D*_ gave more variety of predictions than the *Q*_*R*_ on CO_2_ emissions relative to gross domestic product and amounts of electricity produced. The relationships are not necessarily linear and model free. We expect that the predictions from *Q*_*D*_ may be more reasonable. The predictions may benefit prevention of further damages of CO_2_ emissions to the environment.

## Conclusions

After the above studies, we can conclude:

1. This paper proposes a new direct nonparametric quantile regression method which is model free. It uses nonparametric density estimation and nonparametric regression techniques to estimate high conditional quantiles. The paper provides a computational five-step algorithm which overcomes the limitations of the estimation in the linear quantile regression model and some other nonparametric quantile regression methods.

2. The Monte Carlo simulation works on the second kind of Gumbel’s bivariate exponential distribution which has a nonlinear conditional quantile function. The simulation is different from the bivariate Pareto distribution which has a linear conditional quantile function, in Huang and Nguyen (2017). The simulation results confirm that the proposed new method is more efficient relative to the regular quantile regression estimators and a local polynomial nonparametric estimator.

3. The proposed new direct nonparametric quantile regression can be used to predict extreme values of snowfall and CO_2_ emission examples in Huang and Nguyen (2017). The proposed direct quantile regression *Q*_*D*_ estimator gives a variety of predictions which fits data very well. The prediction of relationships are not simply just linear. We expect that the predictions from *Q*_*D*_ may be more reasonable than the regular quantile regression predictions. The new estimator may benefit prevention of further damages of the extreme events to human and the environment.

4. The proposed direct nonparametric quantile regression provides an alternative way for quantile regression. Further studies on the details of this method are suggested.
